# A comprehensive analysis of gene expression profiling data in COVID-19 patients for discovery of specific and differential blood biomarker signatures

**DOI:** 10.1038/s41598-023-32268-2

**Published:** 2023-04-05

**Authors:** Maryam Momeni, Maryam Rashidifar, Farinaz Hosseini Balam, Amir Roointan, Alieh Gholaminejad

**Affiliations:** 1 Department of Biotechnology, Faculty of Biological Science and Technology, The University of Isfahan, Isfahan, Iran; 2 Department of Plant Sciences and Biotechnology, Faculty of Life Sciences and Biotechnology, Shahid Beheshti University, Tehran, Iran; 3 Department of Cellular and Molecular Nutrition, Faculty of Nutrition and Food Technology, National Nutrition and Food Technology Research Institute, Shahid Beheshti University of Medical Sciences, Tehran, Iran; 4 Regenerative Medicine Research Center, Faculty of Medicine, Isfahan Univerity of Medical Sciences, Hezar Jarib St, Isfahan, 81746-73461 Iran

**Keywords:** Computational biology and bioinformatics, Molecular biology, Systems biology, Biomarkers, Molecular medicine, Pathogenesis

## Abstract

COVID-19 is a newly recognized illness with a predominantly respiratory presentation. Although initial analyses have identified groups of candidate gene biomarkers for the diagnosis of COVID-19, they have yet to identify clinically applicable biomarkers, so we need disease-specific diagnostic biomarkers in biofluid and differential diagnosis in comparison with other infectious diseases. This can further increase knowledge of pathogenesis and help guide treatment. Eight transcriptomic profiles of COVID-19 infected versus control samples from peripheral blood (PB), lung tissue, nasopharyngeal swab and bronchoalveolar lavage fluid (BALF) were considered. In order to find COVID-19 potential Specific Blood Differentially expressed genes (SpeBDs), we implemented a strategy based on finding shared pathways of peripheral blood and the most involved tissues in COVID-19 patients. This step was performed to filter blood DEGs with a role in the shared pathways. Furthermore, nine datasets of the three types of Influenza (H1N1, H3N2, and B) were used for the second step. Potential Differential Blood DEGs of COVID-19 versus Influenza (DifBDs) were found by extracting DEGs involved in only enriched pathways by SpeBDs and not by Influenza DEGs. Then in the third step, a machine learning method (a wrapper feature selection approach supervised by four classifiers of k-NN, Random Forest, SVM, Naïve Bayes) was utilized to narrow down the number of SpeBDs and DifBDs and find the most predictive combination of them to select COVID-19 potential Specific Blood Biomarker Signatures (SpeBBSs) and COVID-19 versus influenza Differential Blood Biomarker Signatures (DifBBSs), respectively. After that, models based on SpeBBSs and DifBBSs and the corresponding algorithms were built to assess their performance on an external dataset. Among all the extracted DEGs from the PB dataset (from common PB pathways with BALF, Lung and Swab), 108 unique SpeBD were obtained. Feature selection using Random Forest outperformed its counterparts and selected IGKC, IGLV3-16 and SRP9 among SpeBDs as SpeBBSs. Validation of the constructed model based on these genes and Random Forest on an external dataset resulted in 93.09% Accuracy. Eighty-three pathways enriched by SpeBDs and not by any of the influenza strains were identified, including 87 DifBDs. Using feature selection by Naive Bayes classifier on DifBDs, FMNL2, IGHV3-23, IGLV2-11 and RPL31 were selected as the most predictable DifBBSs. The constructed model based on these genes and Naive Bayes on an external dataset was validated with 87.2% accuracy. Our study identified several candidate blood biomarkers for a potential specific and differential diagnosis of COVID-19. The proposed biomarkers could be valuable targets for practical investigations to validate their potential.

## Introduction

The novel coronavirus (2019-nCoV, or COVID-19) was first identified at the end of 2019 and has rapidly spread worldwide. It causes severe acute respiratory syndrome and can lead to pneumonia^1^. Detecting and monitoring the disease as early as possible is paramount to preventing progression. COVID-19 shares overlapping signs, symptoms, laboratory findings and imaging features with other respiratory viruses, which might complicate its diagnosis, treatment, and prognosis^2^. Recently, the under-detection of many infectious diseases has increased, which is somewhat due to the prevalence of a novel coronavirus^2^. Influenza, a contagious viral disease-causing respiratory illness, shared similar clinical manifestations to COVID-19. Fever, cough, rhinitis, sore throat, headache, shortness of breath, and myalgia are some of these similar symptoms^3,4^. Different subtypes of the influenza A virus, including H1N1, H3N2, and influenza B as a seasonal influenza virus, are currently circulating among individuals^5^. The co-occurrence of influenza and COVID-19 may increase in the year’s cold months. Both viruses are spread from person to person primarily by airborne droplets^6^. Failures in differential detection of COVID-19 may result in higher hospitalization rates, prolonged stay in intensive care units, and an increased chance of death in patients^7,8^.

Searching for the virus-specific genetic materials via real-time quantitative polymerase chain reaction (RT-qPCR), so far, is the most reliable method for the detection of coronavirus. However, the procedure of RT-qPCR on virus-specific genetic materials is unable to distinguish between active infection and colonization but host-response biomarkers are able to do that^7,9,10^. Furthermore, RT-qPCR can have a high rate of false-negative results due to the low virus load in individuals, which can also change over time, as well as incorrect sampling. This makes it essential to use host-specific biomarkers as a complementary tool to ensure accurate diagnosis of presence or type of infection in at-risk hosts^11–13^.

Numerous tissues, including respiratory epithelial cells, nasopharynx, colonocytes, and whole blood or plasma samples, have recently seen significant changes to the host transcriptome following COVID-19 infection^14,15^. Therefore, transcriptomics can be used effectively to identify COVID-19 affected host transcriptional signatures, paving the way for the creation of novel diagnostic biomarkers and therapeutic strategies^12^. To find virus-specific transcriptional signatures, it is also necessary to comprehend the host response to COVID-19 infection in comparison to other respiratory infections^16^. Although several candidate gene biomarkers have been proposed so far, none of them were successful for an efficient diagnosis and particularly differential diagnosis of COVID-19 in samples.

**Figure 1:**
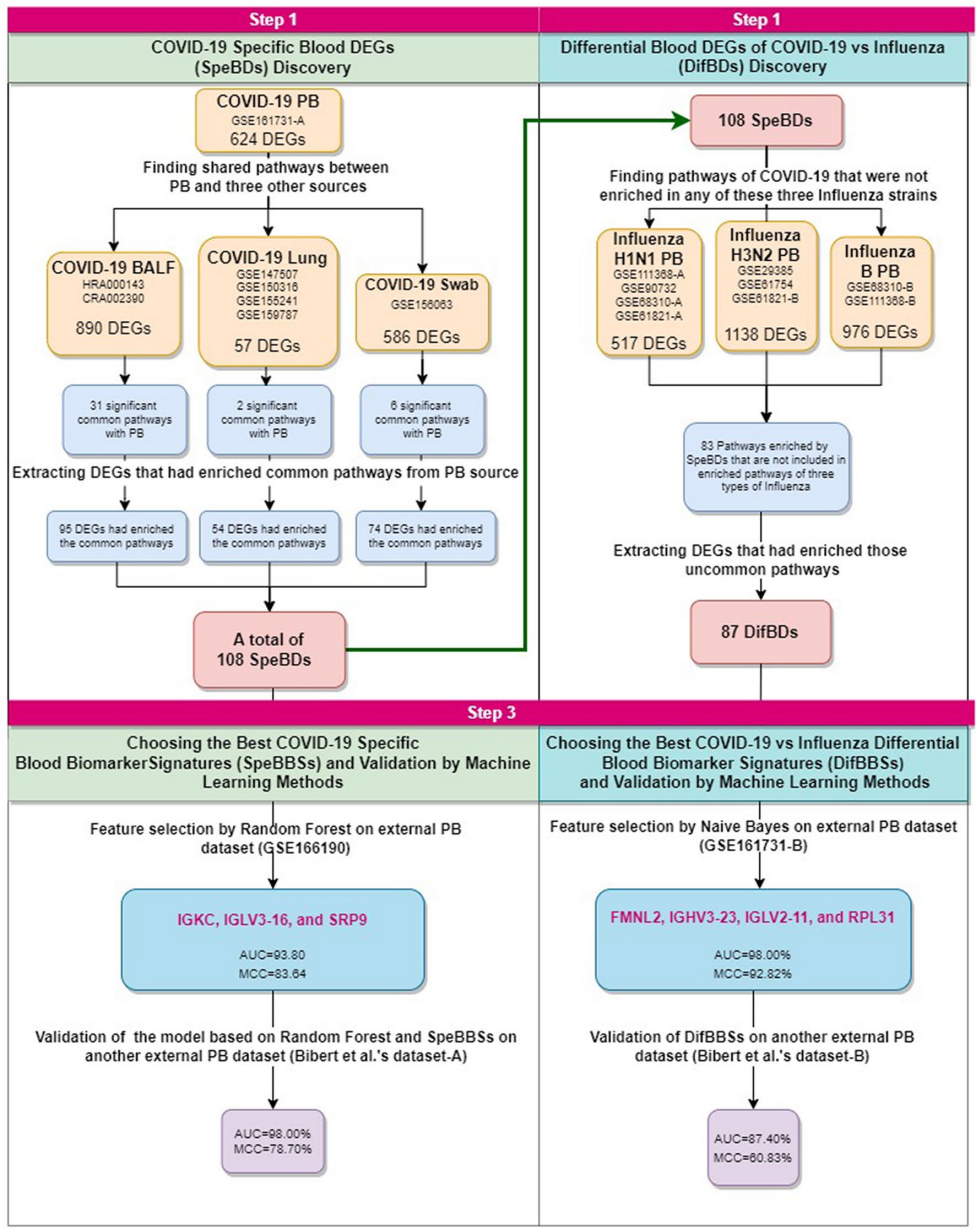
A workflow representing the main steps of the present study. Designed using diagram.net online tool available at https://app.diagrams.net/.

In the present study, we hypothesized that novel and potentially more specific blood biomarkers of a disease could be identified by searching for the DEGs involved in the common pathways between blood and the major organs affected by the disease. We validated this hypothesis using machine learning methods and found that these potential biomarkers included signatures that could accurately differentiate COVID-19 from Influenza blood samples^7^. So, in order to identify COVID-19 potential Specific Blood Differential expressed genes (SpeBDs), we implemented a strategy based on finding shared pathways of peripheral blood (PB) and the most involved tissues in COVID-19 patients (lung tissue, nasopharyngeal swab and bronchoalveolar lavage fluid (BALF)) to filter blood DEGs based on playing a role in those shared pathways. Furthermore, potential Differential Blood DEGs of COVID-19 versus influenza (DifBDs) were identified by extracting DEGs involved in only enriched pathways by SpeBDs and not by influenza DEGs. Then, a machine learning method (feature selection) was utilized to narrow down the number of SpeBDs and DifBDs and find the most predictive combination of DEGs. This step was performed to select potential COVID-19 Specific Blood Biomarker Signatures (SpeBBSs) and COVID-19 versus influenza Differential Blood Biomarker Signatures (DifBBSs), respectively. Then the models based on the SpeBBSs or DifBBSs and the corresponding algorithms were validated on an external dataset. Accuracy (ACC), Area under curve (AUC) and Matthews Correlation Coefficient (MCC) were calculated to measure the power of machine learning models constructed by considering SpeBBSs and DifBBSs. Different steps of this experiment are demonstrated in Fig. 1.

## Materials and methods

### Datasets selection

For finding SpeBDs, transcriptomic profiles of COVID-19 infected versus control samples from PB and three sources related to the respiratory system, the most involved tissues in COVID-19, were considered, including Lung Tissue (Lung), Nasopharyngeal Swab (Swab), and Bronchoalveolar Lavage Fluid (BALF). Datasets of PB, Lung, and Swab sources were obtained from GEO database^17^. Also, the differential expression analysis (DEA) data of the BALF source was obtained from Zhou et al.’s^18^ and Li et al.’s study^19^. In addition, datasets of the three types of Influenza (H1N1, H3N2, and B) were used to discover DifBDs. Table 1 provides all the information about dataset IDs, data production platforms, and sample sizes.

**Table 1:** Publicly available biomarker discovery datasets.

Disease	Sample type^a^	Dataset ID	Technology/platform/platform ID	Data repository^b^	Sample size (I/H)^c^	Data format analyzed in this study
COVID-19	Swab	GSE156063	RNAseq/Illumina NovaSeq 6000/GPL24676	GEO	93/41	Counts
	BALF	HRA000143	RNAseq/Illumina HiSeq 2500	hGSA-BIG	8/20	DEA results of Zhou et al.’s’s study^18^
		CRA002390	RNAseq/Illumina MiSeq	GSA-BIG	4/3	DEA results of Li et al.’s’s study^19^
	Lung	GSE147507	RNAseq/Illumina NextSeq 500/GPL18573	GEO	2/2	Counts
		GSE150316	RNAseq/Illumina NextSeq 500/GPL15520	GEO	41/5	Counts
		GSE155241	RNAseq/Illumina NovaSeq 6000/ GPL24676	GEO	3/2	SRRs
		GSE159787	RNAseq/NextSeq 550 GPL29228	GEO	85/53	Counts
	PB	GSE161731-A	RNAseq/Illumina NovaSeq 6000/GPL24676	GEO	46/19	Counts
Influenza H1N1	PB	GSE111368-A	Microarray/Illumina/GPL10558	GEO	154/131	Series matrix
		GSE90732	Microarray/Illumina/GPL10558	GEO	86/22	Series matrix
		GSE68310-A	Microarray/Illumina/GPL10558	GEO	166/43	Series matrix
		GSE61821-A	Microarray/Illumina/GPL10558	GEO	86/0	Series matrix
Influenza H3N2	PB	GSE61754	Microarray/Illumina/GPL10558	GEO	16/17	Series matrix
		GSE29385	Microarray/Illumina/GPL10558	GEO	36/225	Series matrix
		GSE61821-B	Microarray/Illumina/GPL10558	GEO	16/0	Series matrix
InfluenzB	PB	GSE111368-B	Microarray/Illumina/GPL10558	GEO	16/130	Series matrix
		GSE68310-B	Microarray/Illumina/ GPL10558	GEO	16/4	Series matrix

^a^Swab: nasopharyngeal swab; BALF: bronchoalveolar lavage fluid; PB: peripheral blood cells; ^b^GSA-BIG/hGSA-BIG, Genome Sequence Archive (GSA)/ Human Genome Sequence Archive (hGSA) in National Genomics Data Center, Beijing Institute of Genomics (BIG), Chinese Academy of Sciences https://bigd.big.ac.cn/gsa-human/; GEO: Gene Expression Omnibus; ArrayExpress, ArrayExpress Archive of Functional Genomics Data https://www.ebi.ac.uk/arrayexpress/. ^c^(I/H), samples from infected patients/samples from healthy controls.

### Differential expression analysis

Among RNAseq datasets of COVID-19, GEO raw data of GSE155241 (Table 1) were analyzed by the Galaxy web server (https://usegalaxy.org/)^20^. Quality control was executed with FastQC (version 0.11.8). The reads were aligned to the human reference genome file (Gencode, release 32, hg38 https://www.gencodegenes.org/human/releases.html) using HISAT2 (version 2.1.0) with default parameters. The reads mapped to the human reference genome were counted using featureCounts Galaxy Version 2.0.1 and default parameters.

The RNAseq count files of this study and all other RNAseq datasets of COVID-19 (Table 1) were analyzed by the following methodology: Bioconductor’s DeSeq2 package was used to identify DEGs from the normalized expression dataset. It was then applied to mine statistically significant DEGs based on the difference in their expression values between samples of the COVID-19 versus control. DEGs with |log2FC|≥ 1 and adjusted *p*-value ≤ 0.05 were considered to be significantly differentially expressed. Also, the DEA results of COVID-19 and Healthy BALF samples from Zhou et al.’s study and Li et al.’s study were filtered by a |log2FC|≥ 1 and adjusted *p*-value < 0.05. After obtaining the DEGs of the COVID-19 datasets related to the four sources (Swab, BALF, Lung, and PB), the results of DEA of the sources with more than one dataset (BALF and Lung) were integrated using the Venn diagram. While, in the cases of Swab and PB sources, only one dataset was related to each of them, and plotting the Venn diagram was not required.

For three influenza types, we selected microarray raw data (Table 1). Microarray data were pre-processed, merged, and analyzed independently by the R programming language for each influenza type. The series matrixes were downloaded from the GEO database. Quantile normalization and log transformation were performed on datasets. The aggregate function averaged multiple expression values assigned to the same gene symbols. The platform for producing the data in each influenza type was the same GPL, and the source of all samples was peripheral blood; the data was homogenous, so we integrated data for each influenza type independently using the merging method. In order to remove the batch effect between datasets, we performed a batch effect removal using the ComBat function from the SVA package. Finally, DEA between three types of influenza and healthy samples was conducted independently using the Limma package. DEGs with a false discovery rate adjusted *p*-value < 0.05 and |log2FC|≥ 0.4 |were considered significant.

### Biomarker discovery using pathway enrichment analysis

#### SpeBDs discovery

The pathway enrichment analysis by the Reactome database in Enrichr web-based tool^21^ was performed for DEGs of each source (Swab, BALF, Lung, and PB) independently. Enriched pathways with adjusted *p*-value < 0.05 were considered significant. After that, common pathways of each Swab, BALF, and Lung source with PB were found, and the DEGs that had enriched those common pathways in PB were extracted. These DEGs were considered as SpeBDs.

#### DifBDs discovery

In order to find DifBDs, the pathway enrichment analysis for the three types of influenza (H1N1, H3N2, and B) was performed independently by the Reactome database of Enrichr. The pathway enrichment analysis for the SpeBDs was performed as well. A pathway was considered significant if the adjusted p-value was smaller than 0.05. Then, a Venn diagram was constructed including the significant pathways of SpeBDs, H1N1, H3N2, and B. Significant specific pathways of COVID-19 that were not enriched in any of the influenza types were selected. After that, the SpeBDs of COVID-19 that had enriched those pathways were extracted. These DEGs were considered as DifBDs.

### Choosing the best biomarker signatures and validation by machine learning

RapidMiner Studio as a powerful tool for biomarker discovery was registered (version 9.7) and utilized to extract and validate biomarker signature from SpeBDs and DifBDs^22–26^.

In this study a two-step machine learning approach was implemented, first we employed four classifiers (k-NN, Random Forest, SVM, Naïve Bayes) to supervise the wrapper feature selection method and extract the best combination of biomarkers from the feature selection dataset (an external dataset different from discovery datasets but containing SpeBDs or DifBDs). In the next step, the models based on optimal subset of biomarkers and the corresponding algorithms (the same algorithms that were applied in feature selection to select them) were validated on the validation dataset (another external dataset different from discovery and feature selection datasets). The logic behind this strategy was that the algorithm applied to supervise a wrapper method has had the best performance ability for a subset of features, among other probable combination of features. So we can use that algorithm for building a model (biomarker panel) based on the corresponding features (SpeBBSs or DifBBSs) and test the model on an external dataset to validate the model. The purpose of employing four classifiers in this study was to get four subsets of genes and build four models and biomarker panels. In this way, we had the chance to consider four biomarker panels with a high classification power and introduce the best one, as our minimal biomarker panel.

#### Feature selection

A biomarker panel containing a less number of genes would be more practical to test in a clinical assay^27,28^. So, we decided to choose a small set of most predictive biomarker signatures from SpeBDs to be introduced as COVID-19 potential Specific Blood Biomarker Signatures (SpeBBSs) and from DifBDs to be introduced as COVID-19 versus influenza Differential Blood Biomarker Signatures (DifBBSs). In order to do that, we applied a machine learning method (feature selection) using the Optimize Selection (forward selection type) operator implemented in Rapid Miner. The Forward Selection is a kind of wrapper feature selection approach. Here, we employed four classifiers (k-NN, Random Forest, SVM, Naïve Bayes) to supervise the wrapper method and extract the best combination of biomarkers from the feature selection dataset.

The Forward Selection strategy initially uses only one attribute (in our case, each attribute is a SpeBD or DifBD). Additional attributes are added until there is no more performance gain by adding an attribute.

Rapid Miner provides several other methods for feature selection including Brute Force, Evolutionary algorithm, Backward Elimination, and many other methods^29^. The Optimize Selection (Brute Force) operator examines all possible combinations of the attribute sets to select the most relevant attribute. This method is not applicable in the case of high-dimensional data due to its comprehensive examination^30^. The evolutionary algorithm selects the most relevant attributes of the dataset using evolutionary algorithms, e.g. genetic algorithm (GA). Backward Elimination starts with all features and it removes the worst feature in each step^30^. We tried using Optimize Selection (Evolutionary) and Optimize Selection (Backward Elimination) operators of Rapid Miner but these algorithms represented lower performances with the low number of features compared to the Forward Selection strategy. The purpose of feature selection in this study is to select a small set of biomarker signatures because such a panel would be more clinically applicable. We, therefore, chose to use Optimize Selection (Forward Selection) operator that has a higher performance in selecting a small set of biomarker signatures.

#### SpeBBSs discovery and validation

The count values of SpeBDs were extracted from dataset GSE166190 and Bibert et al.’s dataset-A^31^, which included peripheral blood samples of healthy people and COVID-19 infected patients. Table 2 listed the sample size and platform properties of these datasets.

**Table 2:** Datasets used for feature selection and validation of blood biomarker signatures by machine learning methods.

Dataset ID	Platform/platform ID	Sample size and type	Usage in this study
GSE166190	Illumina HiSeq 4000/GPL20301	15 Healthy, 83 COVID-19	Feature selection for finding SpeBBSs
Bibert et al.'s dataset-A	Illumina HiSeq 4000/-	27 Healthy, 103 COVID-19	Validation of SpeBBSs
GSE161731-B	Illumina NovaSeq 6000/GPL24676	17 Influenza, 77 COVID-19	Feature selection for finding DifBBSs
Bibert et al.'s dataset-B	Illumina HiSeq 4000/-	22 Influenza, 103 COVID-19	Validation of DifBBSs

The rlog function of the package DESeq2 was used to convert the raw counts to normalized logarithmic counts. The dataset was then transposed (samples in rows and SpeBDs genes in columns), and after conversion of disease status to binominal (Healthy = 0 and COVID-19 = 1) input dataset for machine learning was prepared. After that, the two-step machine learning procedure was used to narrow down the SpeBDs for obtaining SpeBBSs (feature selection phase using an external dataset (GSE166190)) and validating the SpeBBSs (validation phase using another external dataset (Bibert et al.’s dataset-A)). In each phase, the five indicators (ACC, Spe, Sen, MCC, and AUC) were calculated for the feature selections and models constructed by the four algorithms.

#### DifBBSs discovery and validation

The count values of DifBDs were extracted from dataset GSE161731-B and Bibert et al.’s dataset B^31^, which included peripheral blood samples of Influenza and COVID-19 infected patients. The sample size and platform properties of these datasets are listed in Table 2. In order to construct the input for RapidMiner software, the binominal disease status (Influenza = 0 and COVID-19 = 1) was added to rlog transformed, transposed counts files of the two datasets. The same two-step procedure for selecting and validating the SpeBBSs was applied to select DifBBSs among DifBDs (feature selection phase using an external dataset (GSE161731-B)) and validate them (validation phase using another external dataset (Bibert et al.’s dataset-B)). In each phase, the five indicators (ACC, Spe, Sen, MCC, and AUC) were calculated for the feature selections and constructed models by the four algorithms.

#### Performance evaluation

The ten-fold cross-validation strategy was employed to evaluate the performance of constructed models in this study. In ten-fold cross-validation, the input (samples) is divided into ten equal parts. One of the ten parts is retained as the test data set. The other parts are used as inputs of the training subprocess. Cross-validation is repeated ten times and every time one of the subsets plays the role of the test dataset. The ten results are then averaged to obtain a single result.

The performance of classification was obtained in terms of four common measurements. These measurements were Accuracy (ACC), Sensitivity (Sen), Specificity (Spe), the Mathews correlation coefficient (MCC), and area under the curve (AUC). The first four were calculated using true-positive (TP), true-negative (TN), false-positive (FP), and false-negative (FN) indicators by the formula. And AUC was calculated by plotting a ROC curve.

When datasets are imbalanced in evaluating binary classification problems, MCC gives more information than other measures like accuracy because it considers the balance ratios of the four measures (TP, TN, FP, and FN). The accuracy score can be misleading since it does not fully consider the size of the four classes of measurements in its final calculation. However, we provided this indicator as the most intuitive evaluation metric. The MCC value is between − 1 and 1. 1 is MCC of a model with the best performance. 0 is like a random prediction, and − 1 indicates a complete discrepancy between reality and prediction^25^. Also, we used AUC, which is a standard parameter and a threshold-independent measure. AUC is the area under the ROC curve generated by plotting sensitivity or true positive rate against false positive rate.

The following parameters were set for the four classifiers of this study:

k-NN: K: 5; Weighted vote: true; Measure types: MixedMeasures; Mixed measure: MixedEucideanDistance. Random Forest: Number of trees: 100; Criterion: gain_ratio; Maximal depth: 10; Voting strategy: confidence vote; Guess subset ratio: true. SVM: Kernel type: Dot; C: 0.00; Convergence epsilon: 0.001; Lpos: 1.0; L neg: 1.0; epsilon: 0.0; Epsilon plus: 0.0; epsilon minus: 0.0. Naïve Bayes : Laplace correlation parameter was set to true.

## Results

### Differential expression analysis and biomarker discovery using pathway enrichment analysis

#### SpeBDs discovery

The DEA between COVID-19 and Healthy PB samples of dataset GSE161731-A (Table 1) resulted in 624 DEGs including 271 upregulated and 353 downregulated genes. The pathway enrichment analysis of these up and downregulated DEGs resulted in 113 significant pathways which are listed in Tables S1 and S2.

**Figure 2:**
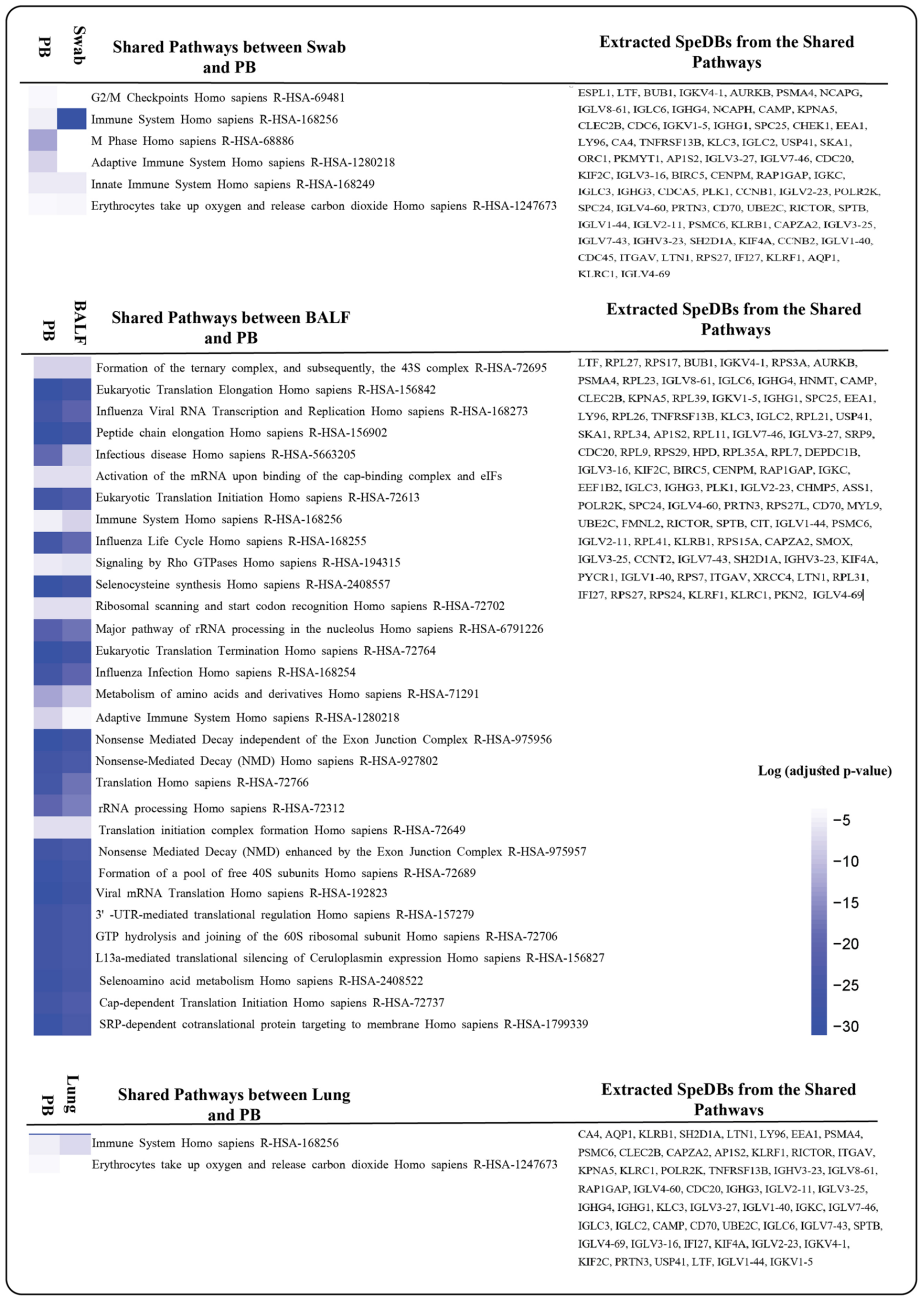
Common Pathways of PB with BALF, Lung, and Swab, their adjusted p-values in pathway enrichment analysis, and the list of extracted SpeBDs from them. The figure is generated using RStudio version 2022.12.0 and Adobe Illustrator version 24.2.1.

**Figure 3:**
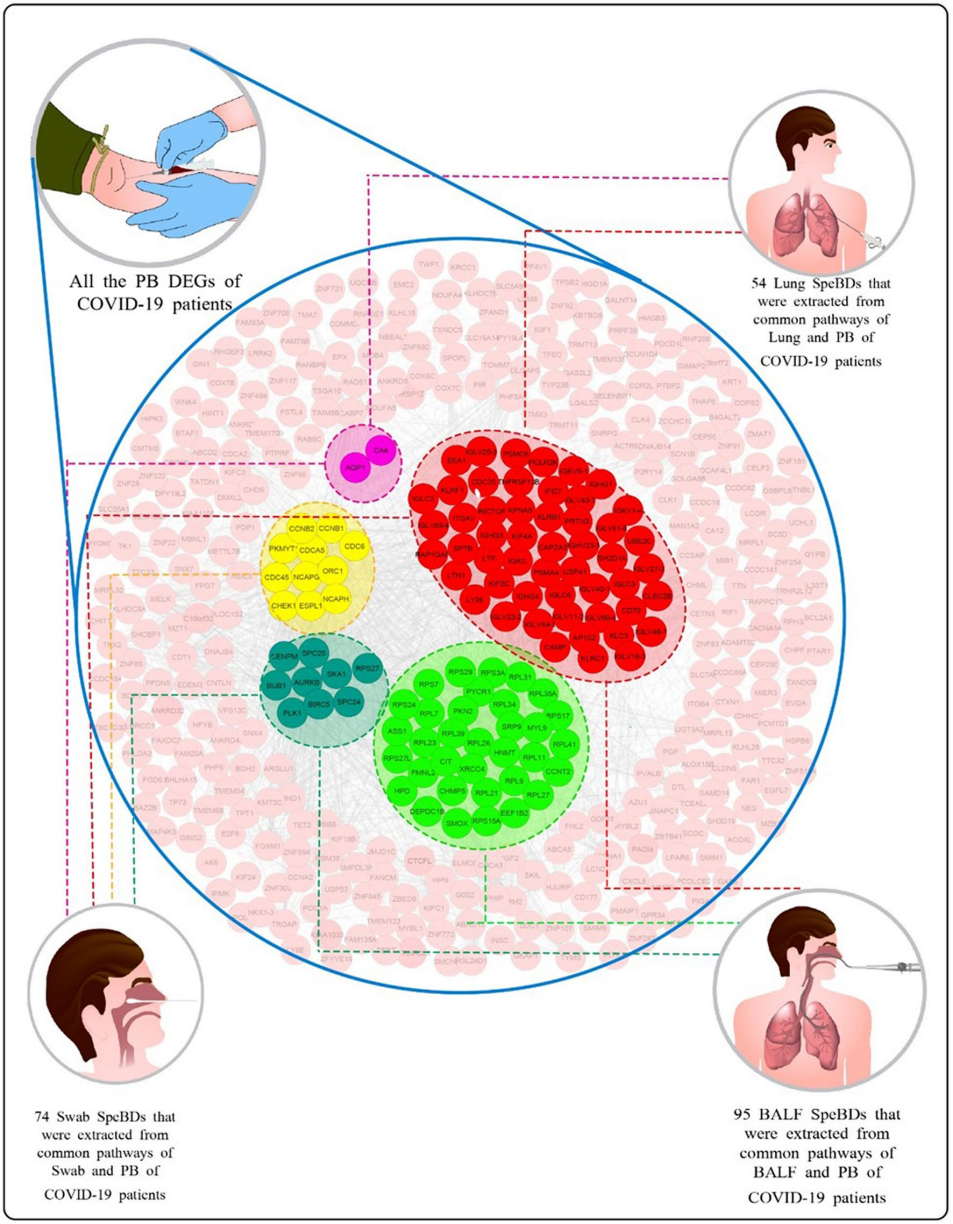
Extraction of SpeBDs from PB DEGs of COVID-19 patients with the help of the common pathways between PB and the three sources from the respiratory system of COVID-19 patients (Swab, BALF, and Lung). A whole list of SpeBDs is indicated in this figure. Lung, Lung tissue biopsy; Swab, nasopharyngeal swab; BALF, bronchoalveolar lavage fluid; PB, peripheral blood. The figure is created using Cytoscape version 3.8.2 and Illustrator version 24.2.1

The DEGs of differential analysis results between COVID-19 and Healthy BALF samples in Zhou et al. and Li et al. studies were obtained. Then, the Venn diagram plotted for these two groups of DEGs from the BALF source resulted in 890 DEGs including 475 upregulated and 415 downregulated genes. The pathway enrichment analysis of these DEGs resulted in 36 significant pathways (Tables S3 and S4). Thirty-one of these significant pathways were shared with the significant pathways of PB, and We extracted 95 DEGs from the PB dataset that had enriched those common pathways (Figs. 2 and 3).

The DEA was performed between COVID-19 and Healthy Lung samples of datasets GSE147507, GSE150316, GSE155241, and GSE159787. The plotted Venn diagram for the four datasets of Lung source resulted in 15 upregulated and 42 downregulated common genes, a total of 57 DEGs. The pathway enrichment analysis of these 57 DEGs resulted in 9 significant pathways (Tables S5 and S6), 2 of these significant pathways were shared with PB, and we extracted 54 DEGs of the PB dataset that had enriched those common pathways (Figs. 2 and 3).

The DEA between COVID-19 and Healthy Swab samples of dataset GSE156063 resulted in 207 upregulated and 379 downregulated genes, a total of 586 DEGs. Pathway enrichment analysis of these DEGs resulted in 91 significant pathways which are listed in Tables S7 and S8; six of which were shared with the PB significant pathways, and 74 DEGs of the PB dataset that enriched those common pathways were extracted (Figs. 2 and 3).

Finally, from all the DEGs extracted from the PB dataset in this step (from common pathways of PB with BALF:95 DEGs, with Lung: 54 DEGs, and with Swab:74 DEGs), duplicated DEGs were removed, and 108 unique SpeBDs were obtained (Fig. 3). A complete list of SpeBDs and their related extraction sources are listed in Figs. 2 and 3. Moreover, a pathway enrichment analysis was performed for the SpeBDs, and 152 significant pathways were enriched (Table S9).

#### DifBDs discovery

In order to obtain DEGs of Influenza H1N1, the four related datasets including GSE111368-A, GSE90732, GSE68310-A, and GSE61821-A were integrated. DEA resulted in 309 upregulated and 208 downregulated, a total of 517 DEGs. These DEGs were enriched in 79 significant pathways (Tables S10 and S11).

To obtain DEGs of Influenza H3N2, three datasets including GSE61754, GSE29385, and GSE61821-B were integrated. The results of DEA were 1139 DEGs including 854 upregulated and 285 downregulated genes. The DEGs were enriched in 11 significant pathways (Tables S12 and S13).

Also, the two datasets of Influenza B (GSE111368-B and GSE68310-B) were integrated, and the DEA resulted in 976 DEGs including 512 upregulated and 464 downregulated genes. The pathway enrichment analysis for these DEGs resulted in 186 significant pathways (Tables S14 and S15).

**Figure 4:**
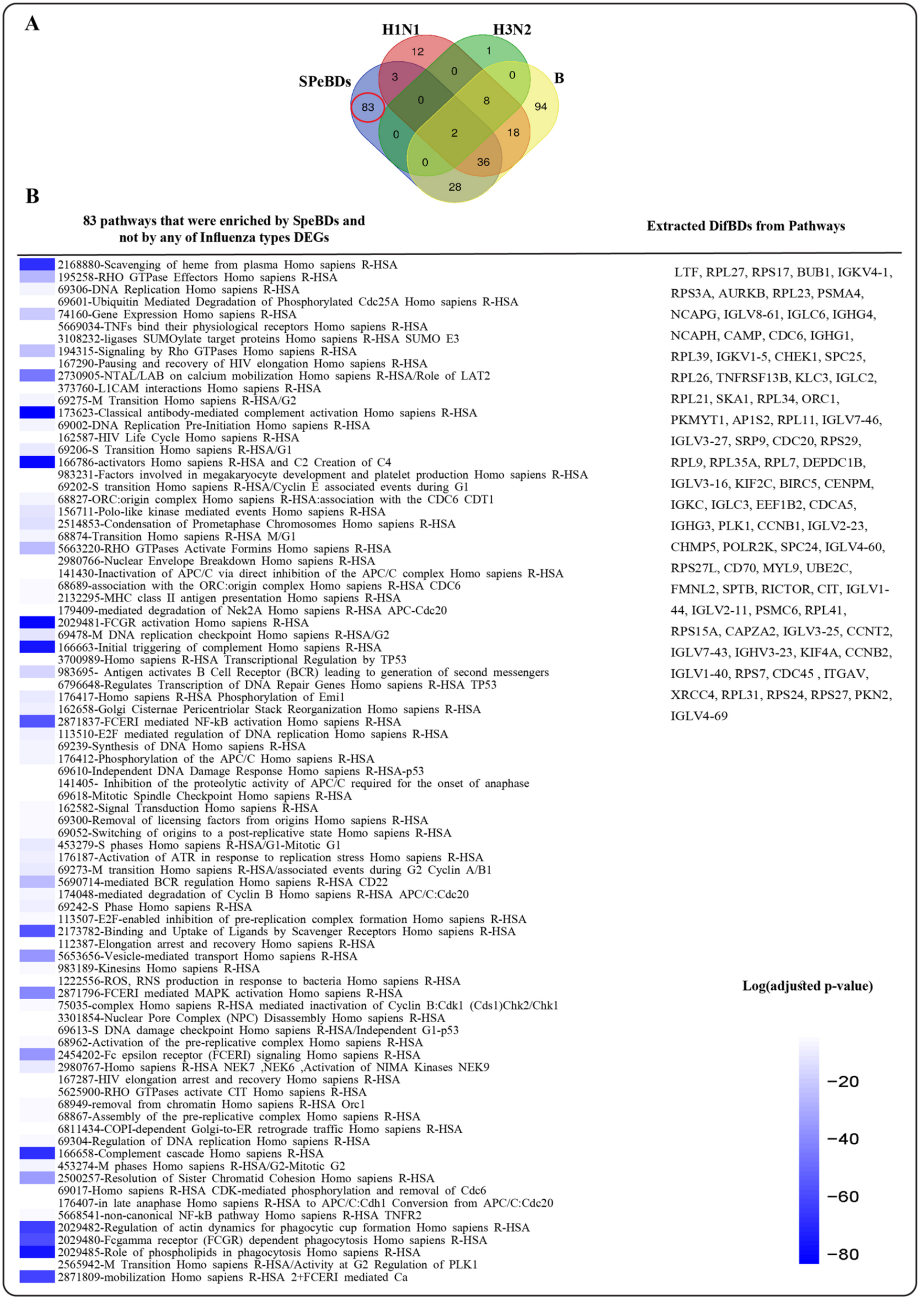
(**A**) Venn diagram representing the pathways enriched by SpeBDs, Influenza H1N1 PB DEGs, Influenza H3N2 PB DEGs, and Influenza B PB DEGs constructed using an online tool available at https://bioinformatics.psb.ugent.be/webtools/Venn/. The red circle mentions pathways that were enriched by SpeBDs and not by the three Influenza types; these pathways are listed in part B: Eighty-three pathways were obtained from pathway enrichment analysis of SpeBDs and were different from pathways obtained by pathway enrichment analysis of Influenza H1N1, H3N2, and B DEGs; (**B**) is created using RStudio version 2022.12.0 and Adobe Illustrator version 24.2.1.

Finally, a Venn diagram of significantly enriched pathways of influenza H1N1, H3N2, B, and SpeBDs was plotted (Fig. 4A). Eighty-three pathways were specifically enriched by SpeBDs and not by any of the Influenza types. The 87 SpeBDs that enriched those pathways were extracted for further analysis and named DifBDs. A list of uncommon pathways and DifBDs from them is provided in Fig. 4B.

### Choosing the best gene signature and validation by machine learning

#### SpeBBSs discovery and validation

**Figure 5:**
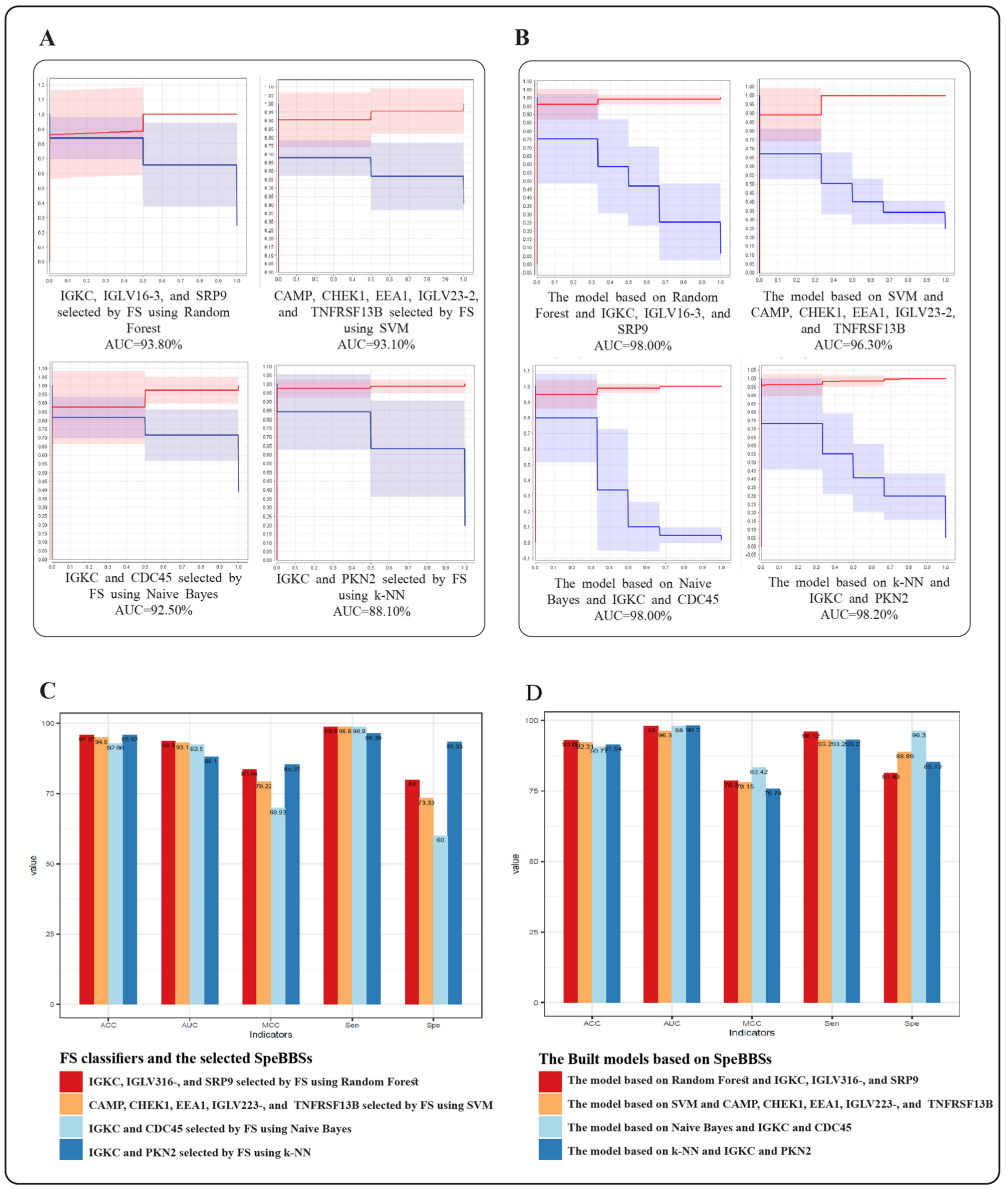
The ten-fold cross-validation results of the feature selection method in choosing SpeBBSs and the constructed machine learning models; (**A**) ROC curves representing classification ability of the feature selection method by the four classifiers on GSE166190 dataset (the feature selection dataset); (**B**) ROC curves representing classification powers of the constructed models based on the selected SpeBBSs and corresponding algorithms (the same algorithms that were applied in feature selection step) on Bibert et al.’s dataset A (the validation dataset). These ROC curves show ROC (red lines) at various threshold settings (blue lines). In the ROC curves, the x-axis shows 1-specificity, and the y-axis shows sensitivity. (**C**) Four measures indicating the classification power of the feature selection method by the four classifiers on GSE166190 dataset (the feature selection dataset); (**D**) Four measures indicating the power of constructed models based on the selected SpeBBSs and the corresponding algorithms (the same algorithms that were applied in feature selection step) on Bibert et al.’s dataset A (the validation dataset). FS: feature selection.

In order to select the best subset of SpeBDs to be introduced as SpeBBSs, a feature selection method was applied using an external dataset containing SpeBDs (GSE166190). Then, these biomarker signatures were validated on another external dataset (Bibert et al.’s dataset -A). All the four classifiers used for evaluating the performance of the feature selection method indicated high robustness levels in terms of AUC and ACC (ACC higher than 92.86% and AUC higher than 86.10% on the feature selection dataset). Also the models based on these algorithms and the SpeBBSs had ACCs higher than 90.77% and AUCs higher than 96.30% on the validation dataset. Feature selection using Random Forest provided the highest ACCs and AUCs (95.92% ACC and 93.80% AUC on feature selection dataset and the model based on this classifier and the three selected SpeBBSs had the 93.09% ACC and 98.00% AUC on the validation dataset) (Fig. 5A,B). Feature selection using this classifier chose IGKC, IGLV3-16, and SRP9 as SpeBBSs. The feature selection and model based on this algorithm had the second-highest performance regarding MCC on both datasets respectively (83.64% on the feature selection and 78.70% on the validation dataset) (Fig. 5C,D). Furthermore, they showed the highest sensitivity with an acceptable level of specificity.

#### DifBBSs discovery and validation

**Figure 6:**
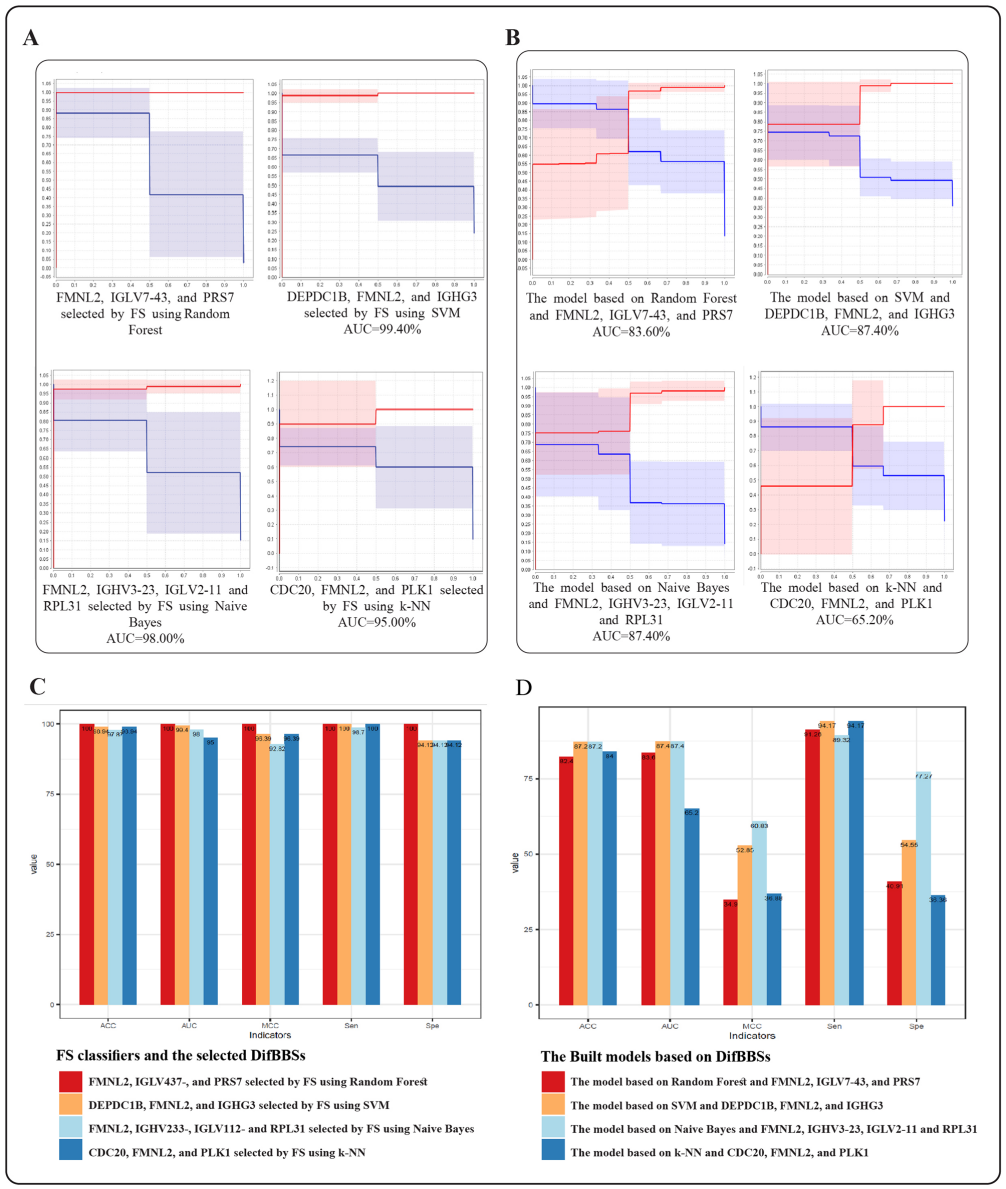
The ten-fold cross-validation results of the feature selection method in choosing DifBBSs and the constructed machine learning models; (**A**) ROC curves representing classification ability of the feature selection method by the four classifiers on GSE161731-B dataset (the feature selection dataset); (**B**) ROC curves representing classification powers of the constructed models based on the selected DifBBSs and corresponding algorithms (the same algorithms that were applied in feature selection step) on Bibert et al.’s dataset-B (the validation dataset). These ROC curves show ROC (red lines) at various threshold settings (blue lines). In the ROC curves, the x-axis shows 1-specificity, and the y-axis shows sensitivity. (**C**) Four measures indicating the classification power of the feature selection method by the four classifiers on GSE161731-B dataset (the feature selection dataset); (**D**) Four measures indicating the power of constructed models based on the selected DifBBSs and the corresponding algorithms (the same algorithms that were applied in feature selection step) on Bibert et al.’s dataset B (the validation dataset). FS: feature selection.

In order to select the most predictable subset of DifBDs to be introduced as DifBBSs, a feature selection method was applied using an external dataset containing DifBDs (GSE161731-B). Then, these biomarker signatures were validated on another external dataset (Bibert et al.’s dataset B). The forward selection method using all four classifiers had ACCs higher than 97.87% and AUCs higher than 95.00% on the feature selection dataset. Models built based on them and the corresponding DifBBSs, represented higher than 82.4 ACCs and higher than 83.60% AUCs on the validation dataset (Fig. 6A,B). Among them, the feature selection using Naive Bayes had a high performance on the feature selection dataset and constructed model based on this classifier and the corresponding DifBBSs represented the highest performance on the validation dataset in terms of MCC; In addition, the feature selection and the model built based on this algorithm showed high levels of sensitivity and specificity in both datasets (Fig. 6C,D). The forward selection method using this classifier chosen FMNL2, IGHV3-23, IGLV2-11, and RPL31 as DifBBSs.

## Discussion

Gene expression profiles of the disease-involved cells are not practical in the diagnosis of diseases. Rather, such profiles might be valuable for selection of limited number of potential protein biomarkers which can be detected via common techniques in biofluid samples. From both basic and clinical perspectives, comprehending the associations between blood biomarkers and the pathogenic states and processes in the tissues affected by the disease could be a great help in selecting the right molecule as potential biomarker. Therefore, in this study, we considered the overlapping pathways between peripheral blood and the central involved body system in COVID-19 in order to identify the disease’s novel and potential specific blood biomarkers^7^. Although, further steps such as comparisons of DEGs of a disease against other diseases (e.g. what we did for Influenza in this study) are indeed needed to get specific biomarkers for diseases, this strategy can help to find the potential specific blood biomarkers before comparing the DEGs of our desired disease against the rest of the diseases one by one. SpeBDs were extracted from the overlapping pathways between PB and respiratory system-related samples (Swab, BALF, and Lung) of Covid-19 patients. The extracted 108 SpeBDs enriched 152 significant pathways that, as we expected, are involved in multiple pathways in the immune system, such as classical antibody-mediated complement activation, FCGR activation, creation of C4 and C2 activators, initial triggering of complement, role of phospholipids in phagocytosis, complement cascade, regulation of actin dynamics for phagocytic cup formation, immune System, immunoregulatory interactions between a Lymphoid and a non-Lymphoid cell, FCERI mediated NF-kB activation, viral mRNA Translation, FCERI mediated Ca + 2 mobilization^32,33^.

In the next step, a machine learning method (feature selection) was utilized to narrow down the number of SpeBDs and find the most predictive combination of them to select SpeBBSs. The five indicators (ACC, AUC, MCC, Sen and Spe) were calculated to measure the power of machine learning models constructed by SpeBBSs. Consequently, feature selection using Random Forest selected IGKC, IGLV3-16, and SRP9 as SpeBBSs with the highest classification power. And the constructed model based on this algorithm and SpeBBSs also validated this biomarker panel on an external dataset. Interestingly, the involvement of these biomarker proteins was previously shown by some studies. Immunoglobulin kappa constant, IGKC, encodes the constant domain of kappa-type light chains for antibodies and Immunoglobulin lambda variable 3-16, IGLV3-16, encodes the variable domain of lambda-type light chains of antibodies. Immunologically, plasma cells are responsible for synthesizing antibodies and have been identified as possibly producing virus-neutralizing antibodies in COVID-19^19,34^. Upregulated IGKC and IGLV3-16 expression may be involved in the differentiation of B lymphocytes into immunoglobulins-secreting plasma cells, which could play an important role in the pulmonary immune response^35^. SRP9 is a component of the signal recognition particle (SRP) complex, involved in targeting secretory proteins to the rough endoplasmic reticulum membrane^35^. The SRP proteins also have a role in the virus-host responses. Based on an experiment, the 7SL RNA component of the SRP interacts with SARS-CoV-2, and upon binding, the viral proteins disrupt SRPs function, thus inhibiting protein trafficking to the cell membrane^36^. Moreover, it was shown that the uncleaved SRP9 could increase the translation elongation arrest and allows translocation, including the insertion of transmembrane domains (e.g., Coronavirus envelope protein). This process can finally lead to frameshifts in the translation process^37^.

In the next part, another pathway-based strategy was applied to obtain DifBDs. 87 DifBDs were extracted from the 83 pathways enriched by SpeBDs but not by Influenza H1N1, H3N2, and B DEGs. The most important of these pathways involves classical antibody-mediated complement activation, FCGR activation, activators, initial triggering of complement, FCERI mediated NF-kB activation, binding and Uptake of Ligands by Scavenger, complement cascade, regulation of actin dynamics for phagocytic cup formation, role of phospholipids in phagocytosis and mobilization. It can be seen that a number of non-specific pathways have been removed from the previous 152 pathways.

Then, DifBBSs were selected from 87 DifBDs using a feature selection approach. The five indicators of ACC, AUC, MCC, Sen and Spe were calculated to measure the power of machine learning methods and models constructed by DifBBSs. Accordingly, the feature selection by the best classifier (the Naive Bayes) selected FMNL2, IGHV3-23, IGLV2-11, and RPL31 as DifBBSs. These DifBBSs along with the Naive Bayes were validated on an external dataset as a biomarker panel with the highest performance. Formin-like protein 2, FMNL2, is a formin-related protein from a family of large proteins with multidomain that play an essential role in controlling a cytoskeletal organization^38^. There is a significant interaction between the native β1 integrins expressed on human and mouse pulmonary epithelial cells and the S-protein of SARS-CoV-2^39,40^. The critical role of β1 integrins in mediating cellular adhesive interaction with the SARS-CoV-2 S-protein have recently shown in studies^39^. As FMNL2 involves in the regulation of β1-integrin traffic and function^41^, it is possible that as COVID-19 progress, FMNL2 regulation shifts from cell-to-cell adhesion to cell-to-substitute adhesion.

IGHV3-23 (Immunoglobulin Heavy Variable 3-23) and IGLV2-11 (Immunoglobulin Lambda Variable 2-11) belong to a cluster of genes in the immunoglobulin (Ig) structure. During acute phase infection in COVID-19, these two variable chains are parts of top frequent paired heavy and light chain clonotypes that are identified in the repertoire of more general clonotypes^42–44^. RPL31 (Ribosomal Protein L31) is a member of ribosomal proteins (RPs). One direct evidence of ribosomal heterogeneity comes from ribosomopathy, caused by defective RPs and/or rRNAs. In a study, the putative role of ribosomal heterogeneity in COVID-19 susceptibility and severity is investigated as an important role^45^. Furthermore, recent studies showed RPL31 as a diagnostic biomarker for this infection^8^.

Conducting the pathway analyses based on a manually curated aggregate of multiple data sources can be the limitation of the present work. On the other hand, the reliability of the findings is maintained by a promise with known mechanisms and between the expression profiling data from different datasets.

## Conclusion

In summary, to find potential specific biomarkers for diagnosis of COVID-19, we focused on disease pathways, which include multiple pathways that can vary between different disease-related compartments. Consequently, more works that simultaneously analyze multiple mechanisms in peripheral blood and inflamed tissues are required. By the way, our findings shed a light on some pathways and molecules which can be valuable candidates for more investigations. Moreover, investigating differential biological pathways in similar diseases can help us identify differential diagnostic biomarkers for diseases. The present study identified several candidate biomarkers for specific detection of COVID-19 and differential diagnosis compared to influenza strains in blood. Further practical studies are necessary to validate these combinatorial biomarkers.

## Supplementary Information

Supplementary Information.

## Data Availability

Data from no human is directly involved in the present study's analysis. All the original data are available in public databases or supplementary material of a published article on the following links: [GEO] repository, [https://www.ncbi.nlm.nih.gov/geo/query/acc.cgi?acc=GSE156063], [hGSA-BIG] repository, [https://ngdc.cncb.ac.cn/gsa-human/browse/HRA000143], [GSA-BIG] repository, [https://ngdc.cncb.ac.cn/gsa/browse/CRA002390], [GEO] repository, [https://www.ncbi.nlm.nih.gov/geo/query/acc.cgi?acc=GSE147507], [GEO] repository, [https://www.ncbi.nlm.nih.gov/geo/query/acc.cgi?acc=GSE150316], [GEO] repository, [https://www.ncbi.nlm.nih.gov/geo/query/acc.cgi?acc=GSE155241], [GEO] repository, [https://www.ncbi.nlm.nih.gov/geo/query/acc.cgi?acc=GSE159787], [GEO] repository, [https://www.ncbi.nlm.nih.gov/geo/query/acc.cgi?acc=GSE161731], [GEO] repository, [https://www.ncbi.nlm.nih.gov/geo/query/acc.cgi?acc=GSE111368], [GEO] repository, [https://www.ncbi.nlm.nih.gov/geo/query/acc.cgi?acc=GSE90732], [GEO] repository, [https://www.ncbi.nlm.nih.gov/geo/query/acc.cgi?acc=GSE68310], [GEO] repository, [https://www.ncbi.nlm.nih.gov/geo/query/acc.cgi?acc=GSE61821], [GEO] repository, [https://www.ncbi.nlm.nih.gov/geo/query/acc.cgi?acc=GSE61754], [GEO] repository, [https://www.ncbi.nlm.nih.gov/geo/query/acc.cgi?acc=GSE29385], [GEO] repository, [https://www.ncbi.nlm.nih.gov/geo/query/acc.cgi?acc=GSE166190], [Bibert et al.'s study supplementary material], https://www.frontiersin.org/articles/10.3389/fimmu.2021.666163/full].

## Abbreviations

SpeBDs: COVID-19 potential Specific Blood DEGs
DifBDs: Potential Differential Blood DEGs of COVID-19 versus Influenza
SpeBBSs: COVID-19 potential specific Blood biomarker signatures
DifBBSs: COVID-19 versus Influenza Differential Blood Biomarker Signatures
DEA: Differential Expression Analysis
DEGs: Differentially Expressed Genes
BALF: Bronchoalveolar Lavage Fluid
PB: Peripheral Blood

## References

[1] Al-Awwal N, Dweik F, Mahdi S, El-Dweik M, Anderson SH (2022). A review of SARS-CoV-2 disease (COVID-19): Pandemic in our time. Pathogens..

[2] Kim D, Quinn J, Pinsky B, Shah NH, Brown I (2020). Rates of co-infection between SARS-CoV-2 and other respiratory pathogens. JAMA.

[3] Dadashi M, Khaleghnejad S, Abedi Elkhichi P, Goudarzi M, Goudarzi H, Taghavi A (2021). COVID-19 and influenza co-infection: A systematic review and meta-analysis. Front. Med..

[4] Zhu, N., Zhang, D., Wang, W., Li, X., Yang, B. & Song, J. *et al.* A novel coronavirus from patients with pneumonia in China, 2019. *N. Engl. J. Med.* (2020). 10.1056/NEJMoa2001017 PMC7092803 31978945

[5] Huang SS, Banner D, Fang Y, Ng DC, Kanagasabai T, Kelvin DJ (2011). Comparative analyses of pandemic H1N1 and seasonal H1N1, H3N2, and influenza B infections depict distinct clinical pictures in ferrets. PLoS ONE.

[6] Kiseleva I, Ksenafontov A (2021). COVID-19 shuts doors to flu but keeps them open to rhinoviruses. Biology..

[7] McClain, M.T., Constantine, F.J., Nicholson, B.P., Nichols, M., Burke, T.W. & Henao, R. *et al.* A blood-based host gene expression assay for early detection of respiratory viral infection: an index-cluster prospective cohort study. The Lancet Infectious Diseases. 2020. 10.1016/S1473-3099(20)30486-2 PMC7515566 32979932

[8] Tang BM, Shojaei M, Parnell GP, Huang S, Nalos M, Teoh S (2017). A novel immune biomarker IFI27 discriminates between influenza and bacteria in patients with suspected respiratory infection. Eur. Respir. J..

[9] Yang, W.E., Woods, C.W. & Tsalik, E.L. in *Methods in Microbiology*, Vol. 42 465–500 (Elsevier, 2015).

[10] Tang, B.M., Shojaei, M., Parnell, G.P., Huang, S., Nalos, M. & Teoh, S. *et al.* A novel immune biomarker IFI27 discriminates between influenza and bacteria in patients with suspected respiratory infection. *Eur. Respir. J.* **49** (6), (2017). 10.1183/13993003.02098-2016 28619954

[11] Maleknia S, Tavassolifar MJ, Mottaghitalab F, Zali MR, Meyfour A (2022). Identifying novel host-based diagnostic biomarker panels for COVID-19: A whole-blood/nasopharyngeal transcriptome meta-analysis. Mol. Med..

[12] Ng DL, Granados AC, Santos YA, Servellita V, Goldgof GM, Meydan C (2021). A diagnostic host response biosignature for COVID-19 from RNA profiling of nasal swabs and blood. Sci. Adv..

[13] Ravichandran S, Banerjee U, Gayathri Devi DR, Kandukuru R, Thakur C, Chakravortty D (2021). VB10, a new blood biomarker for differential diagnosis and recovery monitoring of acute viral and bacterial infections. EBioMedicine.

[14] Ong EZ, Chan YFZ, Leong WY, Lee NMY, Kalimuddin S, Mohideen SMH (2020). A dynamic immune response shapes COVID-19 progression. Cell Host Microbe.

[15] Liao M, Liu Y, Yuan J, Wen Y, Xu G, Zhao J (2020). Single-cell landscape of bronchoalveolar immune cells in patients with COVID-19. Nat. Med..

[16] Oliviero A, de Castro F, Coperchini F, Chiovato L, Rotondi M (2021). COVID-19 pulmonary and olfactory dysfunctions: is the chemokine CXCL10 the common denominator?. Neuroscientist.

[17] Edgar R, Domrachev M, Lash AE (2002). Gene expression omnibus: NCBI gene expression and hybridization array data repository. Nucleic Acids Res..

[18] Zhou Z, Ren L, Zhang L, Zhong J, Xiao Y, Jia Z (2020). Heightened innate immune responses in the respiratory tract of COVID-19 patients. Cell Host Microbe.

[19] Li G, Wang J, He X, Zhang L, Ran Q, Xiong A (2020). An integrative analysis identifying transcriptional features and key genes involved in COVID-19. Epigenomics.

[20] Afgan E, Baker D, Batut B, Van Den Beek M, Bouvier D, Čech M (2018). The Galaxy platform for accessible, reproducible and collaborative biomedical analyses: 2018 Update. Nucleic Acids Res..

[21] Xie Z, Bailey A, Kuleshov MV, Clarke DJ, Evangelista JE, Jenkins SL (2021). Gene set knowledge discovery with Enrichr. Curr. Protoc..

[22] Cheng J, Liu H-P, Lin W-Y, Tsai F-J (2020). Identification of contributing genes of Huntington’s disease by machine learning. BMC Med. Genomics.

[23] Buchanan DM, Ros T, Nahas R (2021). Elevated and slowed EEG oscillations in patients with post-concussive syndrome and chronic pain following a motor vehicle collision. Brain Sci..

[24] Aghamaleki, F.S., Mollashahi, B., Nosrati, M., Moradi, A., Sheikhpour, M. & Movafagh, A. Application of an artificial neural network in the diagnosis of chronic lymphocytic leukemia. *Cureus*. 11(2) (2019). 10.7759/cureus.4004 PMC6450593 31001458

[25] Emmens JE, Jones DJ, Cao TH, Chan DC, Romaine SP, Quinn PA (2018). Proteomic diversity of high-density lipoprotein explains its association with clinical outcome in patients with heart failure. Eur. J. Heart Fail..

[26] Troisi J, Cavallo P, Richards S, Symes S, Colucci A, Sarno L (2021). Noninvasive screening for congenital heart defects using a serum metabolomics approach. Prenat. Diagn..

[27] Lee MY, Kim T-K, Walters K-A, Wang K (2019). A biological function based biomarker panel optimization process. Sci. Rep..

[28] Gholaminejad A, Gheisari Y, Jalali S, Roointan A (2021). Comprehensive analysis of IgA nephropathy expression profiles: identification of potential biomarkers and therapeutic agents. BMC Nephrol..

[29] Schowe, B. (eds) Feature selection for high-dimensional data with RapidMiner. In: *Proceedings of the 2nd RapidMiner Community Meeting And Conference (RCOMM 2011)* (Aachen, 2011).

[30] Asgarnezhad, R., Shekofteh, M. & Boroujeni F.Z. Improving diagnosis of diabetes mellitus using combination of preprocessing techniques. *J. Theor. Appl. Inf. Technol.* **95** (13), (2017).

[31] Bibert S, Guex N, Lourenco J, Brahier T, Papadimitriou-Olivgeris M, Damonti L (2021). Transcriptomic signature differences between SARS-CoV-2 and influenza virus infected patients. Front. Immunol..

[32] Merle, N.S., Noe, R., Halbwachs-Mecarelli, L., Fremeaux-Bacchi, V. & Roumenina, L.T. Complement system Part II: Role in immunity. *Front. Immunol.* **6** (2015). 10.3389/fimmu.2015.00257 PMC4443744 26074922

[33] Merle, N.S., Church, S.E., Fremeaux-Bacchi, V. & Roumenina, L.T. Complement system Part I: Molecular mechanisms of activation and regulation. *Front. Immunol.* **6** (2015). 10.3389/fimmu.2015.00262 PMC4451739 26082779

[34] Wen W, Su W, Tang H, Le W, Zhang X, Zheng Y (2020). Immune cell profiling of COVID-19 patients in the recovery stage by single-cell sequencing. Cell Discov..

[35] Agostini C, Semenzato G (1990). Immune responses in the lung: Basic principles. Lung.

[36] Srivastava, M., Hall, D., Omoru, O.B., Gill, H.M., Smith, S. & Janga, S.C. Mutational landscape and interaction of SARS-CoV-2 with host cellular components. *Microorganisms*. **9** (9) (2021). 10.3390/microorganisms9091794 PMC8464733 34576690

[37] Zecha J, Lee CY, Bayer FP, Meng C, Grass V, Zerweck J (2020). Data, reagents, assays and merits of proteomics for SARS-CoV-2 research and testing. Mol. Cell. Proteomics MCP..

[38] Faix J, Grosse R (2006). Staying in shape with formins. Dev. Cell.

[39] Park EJ, Myint PK, Appiah MG, Darkwah S, Caidengbate S, Ito A (2021). The spike glycoprotein of SARS-CoV-2 binds to β1 integrins expressed on the surface of lung epithelial cells. Viruses..

[40] Sigrist CJ, Bridge A, Le Mercier P (2020). A potential role for integrins in host cell entry by SARS-CoV-2. Antivir. Res..

[41] Wang Y, Arjonen A, Pouwels J, Ta H, Pausch P, Bange G (2015). Formin-like 2 promotes β1-integrin trafficking and invasive motility downstream of PKCα. Dev. Cell.

[42] Rao, S., Srivastava, K., Verma, A. & Das A. B cell receptor repertoire analysis unveils dynamic antibody response and severity markers in COVID-19 patients. *bioRxiv* (2022).

[43] He B, Liu S, Wang Y, Xu M, Cai W, Liu J (2021). Rapid isolation and immune profiling of SARS-CoV-2 specific memory B cell in convalescent COVID-19 patients via LIBRA-seq. Signal Transduct. Target Ther..

[44] He B, Liu S, Wang Y, Xu M, Cai W, Liu J (2021). Rapid isolation and immune profiling of SARS-CoV-2 specific memory B cell in convalescent COVID-19 patients via LIBRA-seq. Signal Transduct. Target Ther..

[45] Shiao, Y.H. Promising assays for examining a putative role of ribosomal heterogeneity in COVID-19 susceptibility and severity. *Life (Basel, Switzerland)*. **12** (2) (2022). 10.3390/life12020203 PMC8880406 35207490

